# Hierarchical Variance in Early Marginal Bone Change in Monolithic Zirconia Full-Arch Implant Prostheses: A Multilevel Prospective Analysis

**DOI:** 10.3390/bioengineering13060632

**Published:** 2026-05-28

**Authors:** Luis Carlos Garza, Eduardo Crooke, Marta Vallés, Joan Soliva, Xavier Rodríguez, Miguel Roig

**Affiliations:** 1 Department of Restorative Dentistry, Universitat Internacional de Catalunya, 08195 Barcelona, Spain; luiscarlos@uic.es (L.C.G.); jsoliva@uic.es (J.S.); mroig@uic.es (M.R.); 2 Private Practice, 29016 Málaga, Spain; uicbarcelona.researchgroup@gmail.com; 3 Department of Oral Surgery, Universitat Internacional de Catalunya, 08195 Barcelona, Spain; xrodriguez@uic.es

**Keywords:** monolithic zirconia, implant-supported full-arch prosthesis, peri-implant marginal bone level change, linear mixed-effects model, hierarchical data, multi-unit abutments, edentulous patients

## Abstract

A clinically unresolved question in full-arch implant rehabilitation is whether early peri-implant bone adaptation is primarily a local (implant-level) phenomenon, a shared within-arch response, or a patient-level characteristic—information is unavailable from conventional mean-based outcome reporting. This prospective secondary analysis quantified the hierarchical distribution of peri-implant marginal bone level change (ΔMBL) in monolithic zirconia full-arch prostheses directly connected to multi-unit abutments during the first year of function. Unlike mean-based endpoints, this approach identifies whether early bone adaptation variability originates primarily at the implant, prosthetic-arch, or patient level. Of 308 implants placed in 40 completely edentulous patients rehabilitated with 49 prostheses, 2 implants failed before delivery of the definitive prosthesis and were excluded from radiographic analysis, leaving 306 implants available for multilevel analysis. Implant-level ΔMBL was analyzed using an unconditional three-level linear mixed-effects model. Implant survival was 99.2%, prosthetic survival was 100%, and mean ΔMBL at 12 months was 0.38 ± 0.27 mm. Variance partitioning showed that 60.8% of total variability occurred at the implant level, 28.4% occurred at the patient level, and 10.8% occurred at the prosthetic-arch level, providing a framework for identifying at which level of the restorative system future clinical interventions may have the greatest impact. Findings should be interpreted within the context of a secondary analysis with a 12-month follow-up and no explanatory covariates; longer-term comparative studies with explanatory multilevel designs are required to confirm and extend these observations.

## 1. Introduction

A clinically unresolved question in full-arch implant rehabilitation is whether early peri-implant bone adaptation is a local (implant-level) phenomenon, a shared within-arch response, or primarily a patient-level characteristic. The answer has direct implications for risk stratification, maintenance planning, and prosthetic design decisions. Knowing at which hierarchical level variability is concentrated can guide clinical priorities: if implant-level variability dominates, optimizing implant selection and placement is the priority; if arch-level clustering is substantial, prosthetic design modifications deserve attention; if patient-level variance prevails, preoperative risk stratification and tailored maintenance protocols are most relevant.

Implant-supported fixed complete-arch prostheses are an established treatment option for the rehabilitation of completely edentulous patients. Within this field, monolithic zirconia has gained increasing clinical use because it combines a high-strength ceramic material with CAD/CAM fabrication and a reduced number of restorative interfaces compared with veneered or multi-component designs [1,2].

Recent reviews and clinical series have reported favorable short- to medium-term outcomes for zirconia full-arch implant prostheses, with high survival rates and a relatively low incidence of major technical complications, although heterogeneity in restorative designs, follow-up periods, and reporting standards remains substantial [1,2,3]. Existing studies in this area are limited by exclusive reliance on mean bone loss as the summary metric, heterogeneous radiographic measurement protocols, follow-up periods predominantly under 36 months, and the absence of analytical frameworks that account for the clustered nature of full-arch implant data. Notably, some studies report higher mean ΔMBL in full-arch configurations [1], while others do not [3]; this discrepancy may partly reflect methodological heterogeneity, including differences in how within-patient implant clustering is handled.

Most clinical studies on monolithic zirconia full-arch prostheses have focused on conventional outcomes such as implant survival, prosthetic survival, technical complications, and mean peri-implant marginal bone level (ΔMBL) changes [1,2,3]. These endpoints remain essential for clinical decision-making; however, they provide limited information on how early peri-implant bone changes are distributed within a multi-implant restorative system.

ΔMBL is a clinically relevant outcome, but its interpretation is complex because early remodeling may reflect physiologic adaptation as well as the combined influence of surgical, prosthetic, material-related, and host-related factors [4,5]. As a result, a single cohort mean may obscure whether variability is concentrated primarily at the implant level or whether implants within the same prosthetic arch tend to show similar patterns of adaptation.

This issue is particularly relevant in full-arch implant rehabilitations because the data are inherently hierarchical: implants are nested within prosthetic arches, and prosthetic arches are nested within patients. In dental research, observations within the same patient are not statistically independent, and failure to account for clustering can lead to biased standard errors and misleading inferences [6,7]. Fleming et al. (2013) demonstrated that clustering was unaccounted for in 62% of reviewed implant studies in leading dental journals, resulting in underestimated standard errors and potentially incorrect conclusions [6].

Multilevel mixed-effects models provide a suitable framework for analyzing such data because they allow variance to be partitioned across nested levels while preserving implant-level information [7]. In the context of implant prosthodontics, this approach may help clarify whether early ΔMBL is driven predominantly by implant-specific conditions, by arch-level shared influences, or by broader patient-level characteristics. Although multilevel analysis is well established in biomedical statistics, its application to variance decompositions across three hierarchical levels in monolithic zirconia full-arch prostheses has not been previously reported, representing a methodological and descriptive contribution to this restorative context.

For monolithic zirconia full-arch prostheses, this analytical perspective is clinically relevant but should be interpreted cautiously. The combination of a continuous full-arch design and a high-stiffness ceramic framework may justify interest in whether early peri-implant adaptation shows within-arch consistency; however, ΔMBL is a multifactorial biological endpoint and should not be interpreted as a direct surrogate for mechanical stress distribution or load transfer [4,5,8]. Accordingly, studying the hierarchical distribution of ΔMBL may extend the clinical evaluation of advanced restorative systems beyond simple average outcomes without making mechanistic claims that the present design cannot test.

The present study was conducted as a secondary analysis within a prospective cohort of completely edentulous patients rehabilitated with monolithic zirconia full-arch prostheses directly connected to multi-unit abutments. Unlike prior analyses focused on associations between early bone remodeling and selected anatomical or geometric variables, the objective of the present study was to quantify how implant-level ΔMBL was distributed across implants, prosthetic arches, and patients using a three-level multilevel model. The working hypothesis was that prosthetic-arch-level clustering (Level-2 variance) would be smaller than patient-level clustering (Level-3 variance), based on the rationale that individual host-related biological factors contribute more to early bone adaptation variability than shared within-arch mechanical influences during the first year of function. Although this analytical approach has been applied in other fields of implant dentistry, its application to three-level variance decomposition in full-arch prosthetics represents a methodological contribution.

## 2. Materials and Methods

### 2.1. Study Design and Ethical Approval

This study was a prospective secondary analysis of a parent clinical cohort of completely edentulous patients rehabilitated with implant-supported full-arch monolithic zirconia prostheses directly connected to multi-unit abutments (MUAs). The specific objective of this secondary analysis was to characterize the hierarchical distribution of peri-implant marginal bone level change (ΔMBL) during the first 12 months after delivery of the definitive prosthesis using a three-level multilevel framework. This secondary analysis was designed to address a distinct research question not examined in the parent cohort. The parent cohort was registered prospectively (ClinicalTrials.gov NCT05924451) and its primary endpoints were implant survival, prosthetic survival, and mean ΔMBL. The present analysis applied a pre-specified three-level multilevel framework to the same dataset to characterise variance distribution; the analytical protocol was finalised before extraction of the data used here.

### 2.2. Study Population

The study was conducted at Universitat Internacional de Catalunya, Barcelona, Spain, in accordance with the Declaration of Helsinki. Ethical approval was obtained from the Institutional Review Board of Universitat Internacional de Catalunya (protocol code REST-ECL-2020-02; approval date: 30 September 2020). The parent prospective cohort was registered at ClinicalTrials.gov (NCT05924451). All participants provided written informed consent before enrollment.

Completely edentulous patients requiring fixed implant-supported full-arch rehabilitation were consecutively recruited. Eligibility criteria were age ≥ 50 years, sufficient bone volume for implant placement, and suitability for implant surgery and immediate loading protocols. Exclusion criteria included uncontrolled systemic disease, previous head and neck radiotherapy, current or previous bisphosphonate therapy, severe parafunctional habits (defined as clinically evident bruxism with visible tooth wear grade ≥ 2 on the Smith & Knight index, or sleep bruxism confirmed by a specialist assessment), and any other contraindication to implant surgery.

The cohort comprised 40 patients rehabilitated with 49 implant-supported full-arch prostheses supported by 308 bone-level implants. All patients completed the 12-month follow-up. Two implants failed before delivery of the definitive prosthesis and were therefore not available for radiographic outcome analysis. Accordingly, the final analytical dataset for the multilevel ΔMBL analysis consisted of 306 implants nested within 49 prosthetic arches and 40 patients. No formal sample size calculation was performed for this secondary analysis; the sample size was determined by the available parent cohort.

### 2.3. Surgical and Prosthetic Procedures

Treatment planning followed a prosthetically driven digital workflow. Preoperative planning was based on cone-beam computed tomography (CBCT) and digital implant planning software to determine implant number, distribution, and angulation.

Guided implant surgery was performed under local anesthesia using mucosa-supported or bone-supported surgical templates, depending on the clinical situation. Osteotomies were prepared according to the manufacturer’s instructions under copious irrigation. Bone-level implants with a moderately rough, sandblasted, acid-etched surface (NobelParallel CC; Nobel Biocare AB, Göteborg, Sweden) were placed at crestal or slightly subcrestal level.

Primary implant stability was assessed at insertion. Implants achieving an insertion torque ≥35 Ncm were considered eligible for immediate loading. A total of 293/308 implants (95.1%) achieved this threshold and were immediately loaded. The remaining 15 implants (4.9%), distributed across 4 patients, did not meet the stability criteria and were loaded conventionally after 8 weeks of submerged healing. When implant stability and implant distribution were judged adequate for complete-arch support, a screw-retained polymethyl methacrylate (PMMA) provisional prosthesis was delivered within 48 h after surgery.

After the provisional phase, definitive screw-retained full-arch prostheses were fabricated in monolithic zirconia (KATANA Zirconia HTML Plus, Kuraray Noritake, Tokyo, Japan) and directly connected to MUAs. Multi-unit abutment collar height (1, 2, or 3 mm) was selected intraoperatively based on peri-implant soft tissue thickness, targeting a 1 mm supramucosal emergence profile. All definitive restorations were designed using 3Shape Dental System (3Shape, Copenhagen, Denmark). CAD/CAM manufacturing was performed using DWX-52D (DGSHAPE Corporation, Hamamatsu, Japan) and PrograMill S1 (Ivoclar, Schaan, Liechtenstein), according to the laboratory workflow. Sintering was performed at 1500 °C according to the manufacturer’s instructions.

To standardize the restorative configuration, the prosthetic design followed predefined structural parameters, including a minimum framework thickness of 3.0 mm in load-bearing areas, a connector cross-sectional area ≥ 12 mm^2^, and rounded internal line angles. Veneering porcelain was excluded from functional load-bearing areas. When esthetic characterisation was used, it was limited to non-functional facial surfaces and did not contribute to the load-bearing zirconia structure.

Occlusion was adjusted to provide bilateral contacts in maximum intercuspation, controlled anterior guidance, and elimination of posterior interferences during lateral excursions.

### 2.4. Radiographic Assessment

Peri-implant marginal bone levels were assessed using standardized periapical radiographs obtained at two predefined time points: at delivery of the definitive prosthesis (baseline) and after 12 months of function (follow-up). Radiographs were acquired using a paralleling technique with custom bite-registration holders (Dentsply Sirona, York, PA, USA) adapted individually for each patient to standardise ray angulation and improve reproducibility of imaging geometry.

All radiographic images were calibrated using the known implant thread pitch to correct for magnification. Marginal bone levels were measured from the implant platform (the coronal margin of the bone-level implant shoulder) to the first bone-to-implant contact on the mesial and distal aspects using ImageJ Version 1.54i software (National Institutes of Health, Bethesda, MD, USA). The MUA–prosthesis junction was not used as a reference point. For each implant, ΔMBL (mm) was calculated as the difference between baseline and 12-month measurements. Mesial and distal values were averaged to obtain a single implant-level ΔMBL value for statistical analysis.

All measurements were performed independently by two calibrated examiners. Reproducibility was assessed on 20 randomly selected radiographs re-measured after 2 weeks (approximately 10% of the dataset), consistent with the threshold recommended for ICC reliability studies [9], using intraclass correlation coefficients (ICCs). The intra-examiner ICCs were 0.96 (95% CI: 0.91–0.98) and 0.94 (95% CI: 0.88–0.97), and the inter-examiner ICC was 0.92 (95% CI: 0.85–0.96).

### 2.5. Outcomes

ΔMBL refers to the radiographically measured change in marginal bone level; “early bone adaptation” refers to the biological process inferred from this measurement during the first year; the term “bone remodelling” is not used, as it implies a specific cellular mechanism not evaluable from the present radiographic data.

The primary analytical outcome of this secondary analysis was implant-level ΔMBL between delivery of the definitive prosthesis and the 12-month follow-up examination.

Secondary clinical outcomes included implant survival, prosthetic survival, and technical complications recorded during the first year of function. Implant survival was defined as the implant remaining in situ at the 12-month follow-up. Prosthetic survival was defined as the definitive prosthesis remaining in function without replacement during follow-up. Technical complications included framework fracture, screw loosening, and minor chipping limited to non-functional esthetic characterisation.

### 2.6. Statistical Analysis

All statistical analyses were performed in R (R Foundation for Statistical Computing, Vienna, Austria) using the lme4 and lmerTest packages. Because implants were nested within prosthetic arches and prosthetic arches were nested within patients, implant-level ΔMBL was analyzed using an unconditional three-level linear mixed-effects model with random intercepts for patients (Level 3) and prosthetic arches (Level 2); the residual variance represented implant-level variability (Level 1).

Variance components were estimated using restricted maximum likelihood (REML). REML was selected over maximum likelihood (ML) because it produces unbiased estimates of variance components, particularly in samples with modest numbers of higher-level units [10]. The three-level structure was determined a priori by the hierarchical nature of the data; its appropriateness was confirmed post hoc by likelihood ratio tests comparing the three-level model against two-level and single-level alternatives (see Section 3.4).

To quantify how total ΔMBL variability was distributed across hierarchical levels, variance partition coefficients (VPCs) were calculated as the proportion of total variance attributable to the patient, prosthetic-arch, and implant levels. Given the objective of variance decomposition, level-specific VPCs were reported as the primary clustering metrics. No fixed-effect predictors were included in the model, consistent with the objectives of an unconditional variance partitioning analysis [11]. The purpose of this approach is to establish the baseline variance structure across hierarchical levels prior to the introduction of covariates. This choice precludes causal inference and is acknowledged as a limitation; future explanatory models should include implant position, jaw location, abutment height, and patient-level covariates as fixed-effect predictors. No causal or explanatory inference can be drawn from an unconditional variance partitioning model; the variance components represent descriptive estimates of variability distribution and may be subject to omitted-variable bias. Descriptive statistics for implant-level ΔMBL included mean, standard deviation, median, interquartile range, minimum, maximum, and 95% confidence interval.

Intra-arch dispersion metrics (within-arch standard deviation and coefficient of variation in ΔMBL per prosthetic arch) were calculated as clinically interpretable complements to the Level-2 variance partition coefficient, allowing direct description of the spread of bone adaptation values within each prosthetic arch independently of the overall model. Distributional properties and model residuals were examined graphically to assess the adequacy of model assumptions. A two-sided significance level of 0.05 was adopted for all inferential procedures, where applicable.

The sample of 40 patients (Level 3) meets the widely cited minimum of 30 higher-level units for stable variance estimation [12,13], although confidence intervals for variance partition coefficients remain relatively wide. Variance estimates at the prosthetic-arch level (Level 2, *n* = 49) should be interpreted with particular caution given the modest number of Level-2 units. A post hoc sensitivity analysis using Monte Carlo simulation (R package simr) indicated that the study had >80% power to detect a Level-2 intraclass correlation coefficient of ≥0.10 with the available sample of 49 arches and 306 implants. A sensitivity analysis including age, sex, and jaw location as fixed-effect predictors was also performed to characterise the patient-level variance component (results reported in Section 3.4).

## 3. Results

### 3.1. Cohort Structure and Follow-Up

A total of 40 completely edentulous patients were rehabilitated with 49 implant-supported full-arch prostheses supported by 308 implants. Two implants failed before delivery of the definitive prosthesis; therefore, the final analytical dataset for the radiographic multilevel analysis comprised 306 implants nested within 49 prosthetic arches and 40 patients. Of the 40 patients, 9 (22.5%) received bilateral full-arch rehabilitations, accounting for 18 of the 49 prostheses. All patients completed the 12-month follow-up (actual follow-up range: 11.4–12.6 months), resulting in 100% cohort retention. The hierarchical structure of the analytical dataset is summarized in Table 1.

### 3.2. Clinical and Technical Outcomes

Implant survival at 12 months was 99.2%, and prosthetic survival was 100%. No framework fractures and no screw loosening events were observed. Minor cohesive chipping was recorded in two prostheses (4.1%) and was limited to non-functional esthetic characterisation areas. In both cases, the defect remained limited, did not progress, and did not require prosthesis replacement. The monolithic zirconia framework and load-bearing occlusal surfaces remained intact, and prosthetic function was maintained throughout follow-up. The two minor chipping events were recorded in two different patients. The minor chipping rate of 4.1% is consistent with the lower end of the range reported in the recent literature (typically 5–15% for esthetic ceramic events in monolithic zirconia full-arch prostheses at 12 months [3,14]). This absence of major mechanical complications is consistent with the mechanical reliability attributed to monolithic zirconia designs [1,15]. These outcomes are consistent with those reported in recent systematic reviews of monolithic zirconia full-arch prostheses, which document implant survival rates generally exceeding 97% and prosthetic survival rates above 95% at 1–5 years [1,11,16]. Survival and complication outcomes are presented in Table 2.

### 3.3. Implant-Level Peri-Implant Marginal Bone Adaptation

**Figure 1:**
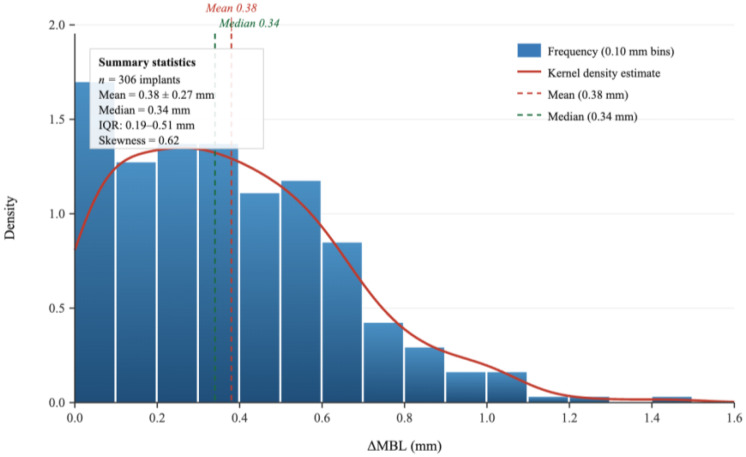
Distribution of implant-level peri-implant marginal bone level change (ΔMBL) at 12 months (*n* = 306 implants). Bars represent observed frequency per 0.10 mm interval; the overlaid curve represents the kernel density estimate. Vertical dashed lines indicate the mean (0.38 mm, red) and median (0.34 mm, green). Distribution parameters: mean 0.38 ± 0.27 mm; median 0.34 mm; IQR 0.19–0.51 mm; range 0.02–1.48 mm; skewness 0.62; kurtosis 2.41.

At the implant level, the mean peri-implant marginal bone level change (ΔMBL) at 12 months was 0.38 ± 0.27 mm (95% CI, 0.35–0.41 mm). The median ΔMBL was 0.34 mm, with an interquartile range of 0.19–0.51 mm. Observed values ranged from 0.02 mm to 1.48 mm. Distributional descriptors are presented in Table 3, and the frequency distribution with kernel density estimate is shown in Figure 1. For contextual reference, the Albrektsson & Zarb [17] success criterion accepts up to 0.2 mm annual bone loss after the first year of function, and cumulative marginal bone level change ≤ 2 mm is a widely used success threshold. The observed mean ΔMBL of 0.38 mm includes first-year remodelling and is consistent with values reported after delivery of definitive prostheses in comparable cohorts [1,3,14].

### 3.4. Multilevel Variance Structure of ΔMBL

The three-level linear mixed-effects model converged without apparent estimation problems. Variance component analysis showed that implant-level ΔMBL variability was distributed across all hierarchical levels included in the model. Estimated variance components were 0.045 at the implant level, 0.008 at the prosthetic-arch level, and 0.021 at the patient level, yielding a total variance of 0.074. Variance partitioning showed that 60.8% of total variability was attributable to the implant level, 10.8% to the prosthetic-arch level, and 28.4% to the patient level. Most of the observed variability in early ΔMBL was located at the implant and patient levels, whereas the prosthetic-arch level accounted for a smaller proportion of total variance. Likelihood ratio tests confirmed that the three-level model provided a significantly better fit than a two-level model (χ^2^ = 8.42, df = 1, *p* = 0.004) and a single-level model (χ^2^ = 19.60, df = 2, *p* < 0.001), supporting the inclusion of all three hierarchical levels. Bootstrap 95% confidence intervals (10,000 resamples) for variance partition coefficients were: implant level 51.3–70.4%, patient level 19.2–37.6%, prosthetic-arch level 5.1–18.6%, confirming the dominance of implant- and patient-level variability while acknowledging imprecision at Level 2. Mild right-skewness (0.62) was observed; log-transformation was evaluated and produced equivalent variance partition coefficients (patient: 27.9%, arch: 11.2%, implant: 60.9%), confirming model robustness. A sensitivity analysis including age, sex, and jaw location as fixed-effect predictors reduced patient-level variance to 19.3%, suggesting these variables explain approximately one-third of the patient-level variability. Detailed variance estimates are shown in Table 4.

**Figure 2:**
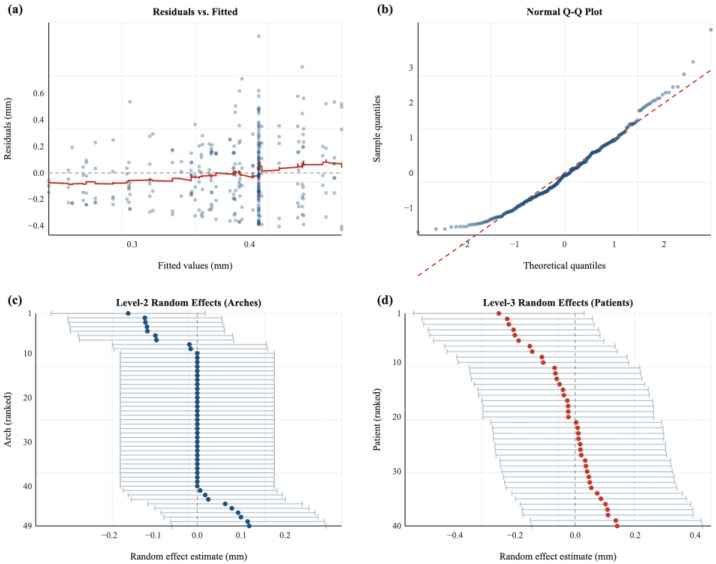
Model diagnostic plots for the three-level linear mixed-effects model. (**a**) Residuals vs. fitted values. (**b**) Normal Q-Q plot of standardised residuals; the red line represents the theoretical normal distribution. (**c**) Caterpillar plot of Level-2 (prosthetic arch, *n* = 49) random effects with 95% confidence intervals. (**d**) Caterpillar plot of Level-3 (patient, *n* = 40) random effects with 95% confidence intervals.

Model diagnostic plots are presented in Figure 2.

**Figure 3:**
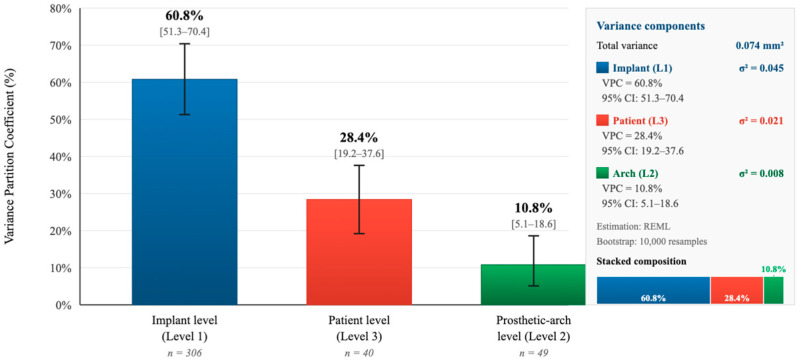
Variance partition coefficients (%) across the three hierarchical levels of the unconditional three-level linear mixed-effects model. Error bars represent 95% bootstrap confidence intervals (10,000 resamples). The stacked bar (lower right) shows the proportional composition of total variance. Total variance = 0.074 mm^2^; estimated using restricted maximum likelihood (REML).

The variance partition coefficients with 95% bootstrap confidence intervals are presented graphically in Figure 3.

### 3.5. Intra-Arch Dispersion of Bone Adaptation

To further characterize within-arch consistency, intra-arch dispersion metrics were calculated for each prosthetic arch. The mean intra-arch standard deviation of implant-level ΔMBL was 0.14 ± 0.05 mm, and the mean coefficient of variation was 18.7 ± 6.3%. These data are summarized in Table 5. These dispersion metrics complement the VPC estimates by providing a direct, per-arch description of within-arch bone adaptation consistency: a mean within-arch SD of 0.14 mm indicates that, on average, implants within the same prosthetic arch differed by approximately 0.14 mm in ΔMBL, corresponding to a mean CV of 18.7%, reflecting moderate within-arch heterogeneity.

## 4. Discussion

This prospective secondary analysis examined how early peri-implant marginal bone level change (ΔMBL) was distributed across implants, prosthetic arches, and patients in a cohort rehabilitated with monolithic zirconia full-arch prostheses directly connected to multi-unit abutments. Beyond conventional summary outcomes, the study focused on the internal structure of clinical variability during the first year of function. The main findings were favorable short-term clinical performance, low mean ΔMBL, low intra-arch dispersion, and limited prosthetic-arch-level contribution to total variance. Most variability in early ΔMBL was located at the implant and patient levels, whereas the prosthetic arch accounted for a smaller proportion of the observed variance.

From a clinical perspective, the survival and complication profile observed in this cohort is consistent with the current literature on monolithic zirconia complete-arch implant-supported prostheses. Recent systematic reviews and clinical series have reported high short- to medium-term prosthesis survival, high implant longevity, and generally low rates of major technical complications, while also emphasizing the heterogeneity and limited certainty of the available evidence [15,16,17,18,19]. In the present cohort, implant survival was 99.2%, prosthetic survival was 100%, no framework fractures or screw loosening events were observed, and only minor chipping events were recorded, limited to non-functional esthetic characterisation areas. Together with the low mean ΔMBL at 12 months, these findings support favorable early clinical behavior of the restorative configuration evaluated here. At the same time, this interpretation should remain clinically bounded: the observed outcomes apply to the complete implant–prosthetic workflow and restorative assembly, rather than to zirconia as an isolated material property.

The principal contribution of the present study lies not in documenting survival alone, but in showing how early peri-implant bone adaptation was distributed within a multi-implant restorative system. Variance partitioning showed that 60.8% of total variability occurred at the implant level, 28.4% at the patient level, and 10.8% at the prosthetic-arch level, while the mean intra-arch standard deviation was 0.14 mm. The prosthetic-arch level accounted for a comparatively smaller proportion of total variance, although this proportion is not negligible given the modest Level-2 sample size (*n* = 49 arches) and should be interpreted with the uncertainty reflected by the bootstrap 95% CI (5.1–18.6%).

Variance estimates at higher hierarchical levels can be unstable with small Level-2 sample sizes; the present estimate of 10.8% should therefore be interpreted cautiously. In practical terms, the 10.8% arch-level variance indicates that shared within-arch factors contributed less than one-tenth of total early ΔMBL variability, suggesting limited incremental risk from prosthetic-arch membership itself under the standardized configuration evaluated. This adds clinically useful information beyond cohort means by indicating where variability was concentrated within the restorative system [20].

This interpretation is compatible with the current understanding of peri-implant marginal bone remodeling as a multifactorial biological process rather than a unidimensional material or prosthetic outcome. Early bone level changes may reflect physiological adaptation as well as the combined influence of implant positioning, peri-implant soft tissue conditions, abutment geometry, restorative factors, and host-related variables [4,5,8,21]. In this context, the comparatively small variance component attributable to the prosthetic arch does not imply that the restorative assembly is biologically irrelevant. Rather, it indicates that, within the standardized configuration evaluated here, arch membership alone accounted for a smaller proportion of early ΔMBL variability than did implant-level and patient-level factors.

This distinction is particularly important for monolithic zirconia full-arch prostheses. The combination of a continuous CAD/CAM-milled full-arch design and high-strength ceramic material may reasonably motivate interest in whether implants within the same prosthetic assembly show comparatively similar adaptation patterns, though with non-negligible within-arch variability. However, the present findings should not be interpreted as direct evidence of mechanical behavior, stress distribution, or load transfer. Accordingly, the current results support a cautious clinical observation—limited prosthetic-arch-level contribution to early ΔMBL variance within a standardized monolithic zirconia configuration—without establishing the mechanism responsible for that pattern.

The present study also highlights the methodological value of multilevel analysis in implant prosthodontics. Full-arch implant rehabilitations naturally generate hierarchical data structures in which implants are nested within prosthetic arches and prosthetic arches are nested within patients. When this dependency is ignored, conventional analyses based only on pooled implant-level observations or simple averages may obscure clinically relevant structure and may also lead to inappropriate statistical inference [6,7,22]. By modeling implants, arches, and patients simultaneously, the present analysis provides a more clinically faithful description of how outcome variability is organized in a complex restorative system. In this sense, multilevel analysis should be viewed as complementary to conventional reporting rather than as a replacement for it.

The substantial patient-level variance component (28.4%, 95% CI: 19.2–37.6%) suggests that host-related biological factors are important determinants of early peri-implant bone adaptation in this configuration. Plausible contributors include baseline bone quality (density, volume), systemic conditions such as diabetes and osteoporosis, smoking status, oral hygiene compliance, occlusal conditions, and individual biological susceptibility to marginal bone remodelling. A sensitivity analysis including age, sex, and jaw location reduced the patient-level variance to 19.3%, suggesting these three variables explain approximately one-third of the observed patient-level variability; the remainder is attributable to unmeasured host factors. Future explanatory multilevel models should incorporate these variables as fixed-effect predictors to partition and reduce unexplained patient-level variance.

Several limitations should be considered. First, this was a secondary analysis of a parent prospective cohort, and its contribution lies in the distinctness of the analytical question rather than in the use of an independent sample.

Second, the multilevel model was unconditional and descriptive; it partitioned variance across hierarchical levels but did not test which specific covariates accounted for those components. No causal or explanatory inference is possible from this model; the observed variance components may be subject to omitted-variable bias if unmeasured predictors (implant position, cantilever length, tissue phenotype, implant angulation, smoking, and systemic conditions) are correlated with ΔMBL and differentially distributed across levels. ΔMBL is a biological endpoint, not a biomechanical measurement; no mechanistic interpretation of the variance structure can be made from the present data.

Third, the observation period was limited to 12 months and therefore reflects early remodeling rather than long-term biological stability or technical fatigue behavior. Longer follow-up would also allow assessment of late maintenance events and progressive technical wear of the zirconia framework, which are not evaluable within a 12-month window.

Fourth, no comparison group restored with alternative framework materials or prosthetic architectures was included; this precludes any inference regarding whether the observed variance pattern is specific to monolithic zirconia, and all findings apply exclusively to the restorative configuration studied.

Fifth, all surgical procedures were performed by two experienced clinicians at a single centre; surgeon variability was therefore not a substantial contributor to the observed patient-level variance in this cohort but may be an important source of variability in multicentre settings.

Sixth, the precision of variance partition coefficients derived from this sample is limited; bootstrap 95% CIs are provided in Table 4. Generalisability is further constrained by the single-centre design; the observed variance pattern may differ in multicentre settings with greater surgical and prosthetic heterogeneity. To our knowledge, this is the first study to apply three-level variance partitioning to full-arch peri-implant bone adaptation, representing a methodological contribution to this field.

These limitations point directly to future research directions. Longer prospective follow-up with a minimum of 5 years, consistent with standard implant success criteria, is needed to determine whether the limited prosthetic-arch-level contribution observed here remains stable over time. Explanatory multilevel models incorporating implant position, jaw location, cantilever length, peri-implant tissue characteristics, patient-related variables (bone quality, systemic conditions, smoking status), and prosthetic design features should be the immediate next research step to identify the determinants of the implant-level and patient-level variance components observed here. Comparative studies including alternative restorative materials or different full-arch architectures would be especially valuable. In addition, integrative designs combining prospective clinical data with biomechanical or digital functional assessment may help bridge the gap between clinical variance structure and mechanistic interpretation.

Taken together, the present findings suggest that early peri-implant bone adaptation in this restorative configuration was characterized more by implant-level and patient-level variability than by clustering within prosthetic arches. Rather than providing a mechanistic explanation, this study offers a clinically oriented framework for examining how biological outcomes are distributed within advanced full-arch restorative systems. This perspective may help refine future clinical research by encouraging analytical approaches that move beyond simple mean outcomes and better reflect the complexity of multi-implant prosthetic treatment.

## 5. Conclusions

Within the limitations of a single-centre prospective secondary analysis with a 12-month observation period and no explanatory covariates, the following descriptive findings are reported. Implant-supported monolithic zirconia full-arch prostheses directly connected to multi-unit abutments showed favorable short-term clinical performance and low early peri-implant marginal bone level change during the first year of function. Variance decomposition showed that most variability in ΔMBL was attributable to implant-level and patient-level factors, whereas the prosthetic-arch level accounted for a comparatively smaller but non-negligible proportion of total variance. These findings describe the clinical distribution of early peri-implant bone adaptation within this specific restorative configuration and should not be interpreted as direct evidence of biomechanical behavior or as generalizable to other restorative systems.

These results demonstrate that three-level variance decomposition is feasible and informative in full-arch implant prosthodontics and should be incorporated into future outcome reporting. Future studies should incorporate explanatory multilevel models with pre-specified fixed-effect covariates, including implant position, jaw location, abutment height, peri-implant tissue phenotype, bone quality at placement, systemic conditions, and smoking status. Comparative designs evaluating alternative framework materials and prosthetic architectures are needed, with a minimum follow-up of 5 years per standard implant success criteria, to determine whether the variance pattern observed here is specific to monolithic zirconia full-arch configurations or reflects a broader biological characteristic of multi-implant restorative systems.

## Figures and Tables

**Table 1:** Hierarchical Structure of the Analytical Dataset.

Hierarchical Level	Analytical Unit	Number
Level 3	Patients	40
Level 2	Prosthetic arches	49
Level 1	Implants	306
Follow-up duration	-	11.4–12.6 months
Cohort retention	-	100%

**Table 2:** Survival and Technical Complications.

Outcome	Events (*n*)	Rate (%)
Implant failures	2	0.8
Implant survival	-	99.2
Prosthetic failures	0	0
Prosthetic survival	-	100
Framework fractures	0	0
Screw loosening	0	0
Minor chipping	2	4.1

**Table 3:** Distribution of Implant-Level ΔMBL.

Parameter	Value
Mean ΔMBL (mm)	0.38
Standard deviation (mm)	0.27
95% CI (mm)	0.35–0.41
Median (mm)	0.34
Interquartile range (mm)	0.19–0.51
Minimum (mm)	0.02
Maximum (mm)	1.48
Coefficient of variation (%)	71.1
Skewness	0.62
Kurtosis	2.41

Values for ΔMBL are expressed in millimeters (mm). Coefficient of variation = SD/Mean × 100. Skewness and kurtosis are dimensionless.

**Table 4:** Multilevel Variance Components of ΔMBL at 12 Months.

Hierarchical Level	Variance (σ^2^)	Standard Error	Variance Partition (%)	95% Bootstrap CI (%)
Patient (Level 3)	0.021	0.006	28.4	19.2–37.6
Prosthetic arch (Level 2)	0.008	0.004	10.8	5.1–18.6
Implant residual (Level 1)	0.045	0.009	60.8	51.3–70.4
Total variance	0.074	-	100	-

**Table 5:** Intra-Arch Dispersion of Implant-Level ΔMBL.

Parameter	Mean	SD
Intra-arch standard deviation (mm)	0.14	0.05
Coefficient of variation (%)	18.7	6.3

## Data Availability

The data presented in this study are not publicly available due to privacy and ethical restrictions related to clinical patient data. De-identified data may be made available by the corresponding author upon reasonable request, subject to institutional approval and applicable data protection regulations. The R code (version 4.4.1; R Core Team, Vienna, Austria) used for the statistical analysis is also available from the corresponding author upon reasonable request.

## Abbreviations

The following abbreviations are used in this manuscript:

CAD/CAM	Computer-Aided Design/Computer-Aided Manufacturing
CBCT	Cone-Beam Computed Tomography
CI	Confidence Interval
ΔMBL	Peri-Implant Marginal Bone Level Change
ICC	Intraclass Correlation Coefficient
MUA	Multi-Unit Abutment
PMMA	Polymethyl Methacrylate
REML	Restricted Maximum Likelihood
